# The impact of disturbed peatlands on river outgassing in Southeast Asia

**DOI:** 10.1038/ncomms10155

**Published:** 2015-12-16

**Authors:** Francisca Wit, Denise Müller, Antje Baum, Thorsten Warneke, Widodo Setiyo Pranowo, Moritz Müller, Tim Rixen

**Affiliations:** 1 Leibniz Center for Tropical Marine Ecology (ZMT), Fahrenheitstrasse 6, 28359 Bremen, Germany; 2 Institute for Environmental Physics, University of Bremen, Otto-Hahn-Allee 1, 28359 Bremen, Germany; 3 Research & Development Center for Marine & Coastal Resources (P3SDLP), Gedung II Balitbang KP Lantai 4, Jalan Pasir Putih II, Ancol Timur, Jakarta 14430, Indonesia; 4 Swinburne University of Technology, Sarawak Campus, Jalan Simpang Tiga, Kuching, Sarawak 93350, Malaysia; 5 Institute of Geology, University of Hamburg, Bundesstrasse 55, 20146 Hamburg, Germany

## Abstract

River outgassing has proven to be an integral part of the carbon cycle. In Southeast Asia, river outgassing quantities are uncertain due to lack of measured data. Here we investigate six rivers in Indonesia and Malaysia, during five expeditions. CO_2_ fluxes from Southeast Asian rivers amount to 66.9±15.7 Tg C per year, of which Indonesia releases 53.9±12.4 Tg C per year. Malaysian rivers emit 6.2±1.6 Tg C per year. These moderate values show that Southeast Asia is not the river outgassing hotspot as would be expected from the carbon-enriched peat soils. This is due to the relatively short residence time of dissolved organic carbon (DOC) in the river, as the peatlands, being the primary source of DOC, are located near the coast. Limitation of bacterial production, due to low pH, oxygen depletion or the refractory nature of DOC, potentially also contributes to moderate CO_2_ fluxes as this decelerates decomposition.

## Abstract (web summary)

Due to increasing disturbance of peatlands, Southeast Asian rivers are thought to play a major role in the transfer of CO_2_ to the atmosphere. Here, the authors present data collected from six Indonesian and Malaysian rivers and show that the region is not an outgassing hotspot as previously assumed.

## Graphical abstract

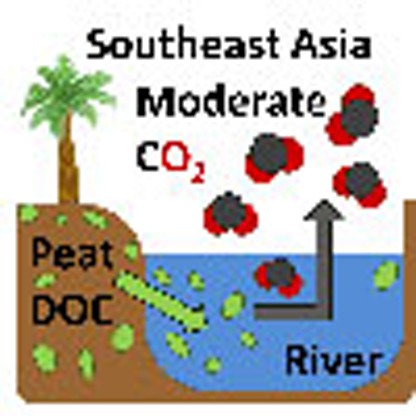

The importance of inland waters in the global carbon cycle has gained more awareness since the last decade through studies which have revealed that inland waters (rivers, streams, lakes, reservoirs and estuaries) are not a passive conduit but play an integral role both for carbon storage and greenhouse gas emissions to the atmosphere^1^,^2^,^3^,^4^. These studies estimate, in line with the 5th Assessment IPCC Report^5^, that on a global scale ∼45–60% (0.9–1.4 Pg C per year) of carbon entering the freshwater system is decomposed and emitted back to the atmosphere as CO_2_. Another 0.2–0.6 Pg C per year is buried in freshwater sediments and about 0.9 Pg C^1^ reaches the coastal ocean. However, an estimate of inland water outgassing by Raymond *et al.*^6^ revealed an emission of 2.1 Pg C per year, of which 1.8 Pg C per year from streams and rivers, which is significantly larger than previous estimates. On the other hand, a more recent study by Lauerwald *et al.*^7^ estimates a global river outgassing of 0.65 Pg C per year, however, they have excluded stream orders <2. These variable findings challenge our current understanding of the global carbon cycle and in particular that of the terrestrial biosphere as a sink for anthropogenic CO_2_. Still, large uncertainties remain in outgassing fluxes due to scarcity of data, which we aim to resolve for Southeast (SE) Asia. Indonesia and Malaysia are areas of particular interest due to their peatlands, which together store 66.5 Pg C (ref. 8). It has been shown recently that the fluvial organic carbon flux increases once these tropical peatlands are disturbed^9^. However, it remains unclear to what extent this influences the CO_2_ emissions from these aquatic systems.

In this study, river outgassing fluxes are quantified for SE-Asia by using measurements from four rivers in Sumatra, Indonesia, and two rivers in Sarawak, Malaysia (Fig. 1). CO_2_ fluxes and yields for all rivers are determined and related to peat coverage, which uncovers a positive relationship. Based on this correlation, CO_2_ fluxes for SE-Asia are calculated, which reveal moderate fluxes and show that SE-Asia is not a hotspot for river outgassing.

## Results

### River processes and variability

In estuaries, salinities higher than zero can be measured 18–50 km downstream with increasing salinities towards the coastal ocean (Fig. 2a), whereas pCO_2_ concentrations show an opposite pattern with increasing concentrations going upstream (Fig. 2c). Indeed, in the estuaries, salinity is inversely correlated with CO_2_ concentrations (Fig. 2b) as a consequence of mixing between river and oceanic waters and the associated decrease of pH in rivers. To calculate mean river parameter values, estuaries were excluded to avoid the influence from ocean waters and only data points with salinity 0–0.1 in the respective rivers were taken into consideration (Fig. 2d, Table 1).

Figure 2d shows the variability of the pCO_2_ data in the Sumatran rivers in 2009 and 2013. The highest pCO_2_ concentrations were measured in the Siak river, which also reveals the highest dissolved organic carbon (DOC) and the lowest O_2_ concentrations (Table 1, Fig. 3). This agrees with results derived from numerical model and DOC decomposition experiments, which show that DOC leaching from peat soils and the decomposition of its labile fraction are the main factors controlling DOC and O_2_ concentrations in the Siak^10^. The resulting input of dissolved inorganic carbon and the already low pH, caused by the organic acids from peat, shift the carbonate system (CO_2_↔
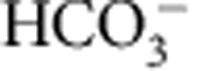
↔
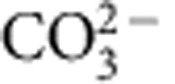
) towards CO_2_ and explain the high CO_2_ concentrations in the Siak. Increasing pCO_2_ concentrations associated with increasing DOC concentrations denote that DOC leaching and decomposition are prime factors controlling pCO_2_ concentrations also in all other studied rivers (Fig. 3).

Contrary to the Siak, which is a typical black-water river where its dark brown colour reduces light penetration to a few centimeters, non-black-water rivers have a higher light availability. Accordingly, photosynthesis plays a role and therefore these rivers have a pronounced day and night cycle, as seen in the Musi river during our expedition in 2013. As we entered this river during the day the pCO_2_ concentrations were lower than during the departure, which took place during the night when photosynthesis and CO_2_ uptake could not take place, but decomposition and CO_2_ production prevailed. In total, the observed variability of pCO_2_ in the Musi was ±21.5%, which was reduced to ±6.2% in the Siak due to a reduced impact of photosynthesis in peat-draining rivers.

The Musi also shows an interannual variability with lower pCO_2_ concentrations in 2009 as compared with 2013 due to lower precipitation rates and lower DOC leaching.

Although this supports our former finding that DOC leaching is a prime factor controlling pCO_2_ concentrations, there are also exceptions, which point to other processes, as indicated by the high pCO_2_ but low DOC concentrations in the Siak in 2013. Contrary to non-peat-draining rivers, enhanced discharge lowers DOC concentrations in rivers draining undisturbed peat. This reverse behaviour is caused by the high water saturation of peat due to which enhanced precipitation leads to flooding and the resulting surface runoff increases discharge, which dilutes the DOC concentrations in the peat-draining rivers^11^,^12^,^13^. However, the peatlands in the Siak catchment as well as elsewhere in Indonesia are largely drained. When precipitation is relatively low, the ground water table could fall below the peat and the DOC concentrations in the river would be reduced^12^. This might explain the exceptionally low DOC concentration in April 2013, which can be associated with low precipitation rates (Table 1). However, despite the low DOC concentration, pCO_2_ concentrations remained high pointing to an additional carbon source. This could have been a decaying plankton bloom favoured by the enhanced light availability due to the lower DOC concentrations. A similar situation might also explain the Lupar in 2013, where low DOC concentrations are associated with low precipitation rates, but relatively high pCO_2_ concentrations still occur. This indicates that the dampening effects of reduced carbon input from soil leaching on the pCO_2_ concentrations could be counterbalanced by input of CO_2_ from presumably plankton blooms, which reduces the temporal variability of pCO_2_ in the rivers during time characterized by extremely low DOC leaching.

### River outgassing and DOC export

Quantification of both river outgassing and riverine DOC export sheds light on their flux ratio and consequently the share of river outgassing with respect to the carbon entering the freshwater system. CO_2_ emissions were calculated for all investigated rivers using equation 1 (Methods section) with their respective piston velocities and respective ΔpCO_2_, which were based on their averaged pCO_2_ values (Table 1) and an atmospheric CO_2_ concentration of 390 μatm. Subsequently, these fluxes were multiplied by their respective river surface area (Methods section and Table 2) to calculate the flux per river. Riverine DOC exports were calculated by multiplying the DOC concentrations with the discharges, which were based on the averaged monthly precipitations (Table 1) and assuming an evapotranspiration of 38% (ref. 9). The resultant net water–air CO_2_ fluxes and riverine DOC exports are presented in Table 3. Note that both CO_2_ and DOC fluxes are shown in grams 10^9^ mth^−1^. These results suggest that on average 53.3±6.5% of the organic carbon leached from soils is decomposed in the river and emitted as CO_2_ into the atmosphere. This percentage is based on monthly averages from six rivers, has a relatively small s.e. and is similar to the 45–60% that was reported as the global average in the IPCC report, as mentioned before. Therefore we may assume that this percentage is also a representative value for river outgassing in SE-Asia.

### CO_2_ fluxes and peat coverage in SE-Asia

As the data of this study is limited to a peat coverage of 21.9%, the lateral DOC exports of 95.7 g C m^−2^ per year for disturbed peat and 61.7 g C m^−2^ per year for pristine peat, as determined in rivers on Borneo by Moore *et al.*^9^, were used to extend the data set to 100% peat coverage. Based on the fact that carbon leaching equals the sum of CO_2_ outgassing and riverine DOC export, this DOC export yield for 100% peat coverage was then converted to CO_2_ yield by assuming a river outgassing of 53.3±6.5% as found in the SE-Asian rivers. This results in CO_2_ yield for 100% peat coverage of 109.2 g C m^−2^ per year (disturbed) and 70.5 g C m^−2^ per year (pristine). Knowing that 90% of SE-Asian peatlands are disturbed and 10% pristine^14^, the CO_2_ yield for 100% peat coverage amounts to 105.3±27.6 g C m^−2^ per year. This ratio for disturbed and pristine is naturally accounted for in the *in situ* river measurements, assuming an even distribution.

The lateral DOC export of 95.7 g C m^−2^ per year and the net ecosystem C loss of disturbed peatlands of 433 g C m^−2^ per year derived from eddy covariance measurements^15^ together amount to 529 g C m^−2^ per year. Including the river outgassing flux of 109.2 g C m^−2^ per year, which accounts for a 20% increase, raises the net ecosystem C loss to a total of 638 g C m^−2^ per year (net C source).

The extent of peat coverage in the investigated rivers differs in each catchment and correlates with the DOC and CO_2_ yield, emphasizing the importance of peat as DOC and CO_2_ source (Fig. 4). The correlation found between peat coverage and CO_2_ yield shows that the correlation initially appears linear, but levels off after a peat coverage of 25%, which indicates that the rate of pCO_2_ production declines with increasing peat coverage. This may be attributed to a limitation of bacterial production as a consequence of the low pH caused by the acidic organic environment^16^ or oxygen depletion (this study, refs 10, 16). Based on the peat coverages and respective CO_2_ yields, the annual CO_2_ fluxes were interpolated for Indonesia, Malaysia and SE-Asia (Table 4) using the regression. As a whole, SE-Asia releases 66.9±15.7 Tg C per year through river outgassing, of which the majority is emitted by Indonesia with 53.9±12.4 Tg C per year. This is due to the fact that Indonesia holds 83% of SE-Asian peatlands in addition to a large land surface area. River outgassing in Malaysia amounts to 6.2±1.6 Tg C per year. Although no CH_4_ measurements were taken in the river, CH_4_ fluxes are not considered to play a significant role in river carbon emissions. This is supported by CH_4_ fluxes from the Saribas and Lupar estuaries, which emit 27±24 t CH_4_–C per year and 84±24 t CH_4_–C per year, respectively, and are a minute fraction of the estuary CO_2_ fluxes 0.09±0.08 and 0.31±0.09 Tg C per year (ref. 17). CH_4_ fluxes from peat soils are with 0.02 g C m^−2^ per year also much lower than those of peat soil CO_2_ fluxes 250 g C m^−2^ per year (ref. 18).

Considering that the data is collected during the transitional stages during the year with respect to precipitation, the mean values represent a suitable yearly average. Although extreme events such as droughts or heavy rainfall have not been measured, their influence will not alter the estimated CO_2_ fluxes significantly. As discussed earlier, the effect of increased discharge due to high water saturated peatlands dilutes the DOC concentration, which dampens the effect of enhanced discharge^11^,^19^,^20^. Therefore a decrease is expected in DOC and hence pCO_2_ concentrations during extreme events in peatland areas. In non-peat areas, enhanced precipitation will increase DOC leaching and hence, DOC and pCO_2_ concentrations in the river waters. However, as seen in Table 4, the impact of peat on the CO_2_ fluxes, wherein the impact is defined as the share of CO_2_ emissions from peatlands expressed in percentage, is much larger than that of non-peat areas. The regression indicates that non-peat areas have a CO_2_ yield of 2.0 g C m^−2^ per year and only contribute 7.1% (4.8 Tg per year) to the CO_2_ fluxes in SE-Asia, as opposed to 92.8% (62.1 Tg per year) from peatlands (Table 3). Assuming a case of heavy rainfall, wherein the CO_2_ yield would double to 4.0 g C m^−2^ per year, would increase the CO_2_ flux from non-peat areas to 9.6 Tg per year, but would not significantly increase the total annual CO_2_ fluxes for SE-Asia. Additionally, the effect of the large peat impact and that CO_2_ fluxes from peatlands will decrease due to dilution from increased discharge, will outweigh the increased CO_2_ flux of non-peat areas, thereby maintaining a steady or even decreased CO_2_ flux during extreme rainfall. Therefore, our annual CO_2_ flux estimates can be assumed to be representative for SE-Asia, wherein fluctuations due to extreme events are accounted for in the error range.

### Study comparison

For data comparison, the CO_2_ flux and piston velocity estimates of Raymond *et al.*^6^ were used, who have calculated global inland water–CO_2_ effluxes by means of a global set of calculated piston velocities, pCO_2_ values^21^ and COSCAT areas (Coastal Segmentation and related CATchments). These COSCAT areas are characterized by their coastal segment limits and length and by catchment characteristics, such as runoff direction and physiographic units^22^. Raymond *et al.*^6^ assigned an average piston velocity and an average pCO_2_ value to each of these COSCATs from which they estimated the CO_2_ yield per m^2^ land per year. Several of these COSCAT areas, 10 of which are relevant for our study area, overlap SE-Asia and are summarized in Table 5. Based on the COSCATs in Table 5 the pCO_2_, K_CO2_ and CO_2_ yield were averaged for Malaysia (row 1–3), Indonesia (row 2–8) and SE-Asia (row 1–10), after which the corresponding CO_2_ flux was calculated and compared with our data (Table 6).

In addition, an indirect comparison was made based on recent estimates by Lauerwald *et al.*^7^ (Table 6). Although no data is available for SE-Asia, they do provide estimates of pCO_2_ concentrations and piston velocities for the tropical zone (<25°), which we used to predict their CO_2_ fluxes in SE-Asia (Table 6), assuming a river surface coverage as determined in our study (Table 2).

The comparison of Raymond *et al.*^6^ with our data reveals that the CO_2_ flux estimates for Indonesia and SE-Asia by Raymond *et al.*^6^ are almost three times higher than those found in this study with 144.7 Tg C per year and 181.5 Tg C per year, respectively. The Malaysian estimate peaks almost eightfold with 48.8 Tg C per year. These overestimations were already anticipated by Raymond *et al.*^6^ and are explained by the small number of calculated CO_2_ values they had available in these COSCATs. Moreover, compared with our findings, their piston velocities appear to be largely overestimated (Table 5) and are mainly responsible for the resulting CO_2_ flux overestimations of Raymond *et al.*^6^. As for Lauerwald *et al.*^7^, their data for the tropical zone (<25°) include piston velocities similar to ours, but show pCO_2_ concentrations that are 21, 40 and 35% lower in Malaysia, Indonesia and SE-Asia, respectively, and result in lower CO_2_ emissions.

### Explanatory arguments for moderate fluxes

The overall statement of this study conveys that SE-Asia is in fact not such a river CO_2_ outgassing hotspot as one could assume due to the carbon-enriched peat soils (Table 4). Even temperate zones, with a CO_2_ yield of 18.5 g C m^−2^ per year (based on an average CO_2_ yield of 2370, g C m^−2^ per year (ref. 23) of river surface area, converted to m^2^ catchment area with a river coverage of 0.78% as estimated for SE-Asia) are close to the CO_2_ yield of 25.2 g C m^−2^ per year of SE-Asia. CO_2_ yields from other tropical river systems, such as the Amazon are much larger with 120±30 g C m^−2^ per year (ref. 24), and show that CO_2_ outgassing from SE-Asian rivers is rather moderate. The main reason for these moderate fluxes is the relatively short residence time^10^ of DOC in the river water due to the location of the peatlands near the coast, which are the main source of DOC. Limitation of bacterial production, as a consequence of the low pH caused by the acidic organic environment^16^, oxygen depletion (this study, refs 10, 16), or the refractory nature of DOC (refs 10, 25, 26), potentially also contributes to the moderate CO_2_ fluxes as this decelerates decomposition, especially with increasing peat coverage.

In conclusion, this study shows that river outgassing fluxes in SE-Asia are in fact moderate and, in line with the 5th Assessment IPCC report, suggest that ∼53.3±6.5% of carbon entering the freshwater system is decomposed and emitted back to the atmosphere as CO_2_. Globally there are three tropical regions, of which Africa^16^ and Amazonia^24^ are significantly important contributors to the river outgassing budget. However, due to the fact that the main source of DOC is located near the coast, which further shortens the residence time of DOC in rivers, SE-Asia is a moderate emitter and global river outgassing estimates can therefore be scaled down. In future assessments, it needs to be considered that rivers function in a different way with respect to discharge, depending on residence time of DOC in the river and the soil type as it strongly affects the DOC leaching and consequently respiration and CO_2_ emission.

## Methods

### Study area

SE-Asian peatlands cover 27.1 million hectares and are located in the coastal plains of the islands of Sumatera, Borneo and Ian Jaya^27^. Tropical peatlands are particularly vulnerable to anthropogenic stressors, such as deforestation and drainage, which are used to convert peat swamp forests into cropland. Today, a large fraction of this peat is already found under oil palm plantations^28^ and only 10% remains undisturbed^14^. The investigated rivers in Sumatra, Indonesia, are the Musi, Batanghari, Indragiri and Siak. All four rivers originate from the Barisan Mountains with a short steep descent and continue to flow towards the Malacca Strait. Towards the river mouths, the rivers cut through peatland areas which scatter the low-lying areas along the northern coast and are subject to leaching of organic matter^29^. The Siak is a typical black-water river^30^; its brown colour is derived from dissolved organic matter leached from adjacent disturbed peatlands, which cover 21.9% of its catchment area. The Indragiri, Batanghari and Musi have a peat coverage of 11.9, 5.0 and 3.5%, respectively^31^. The thickness of the Sumatran peatlands varies between 2 and 10 m (ref. 27). Malaysia has ∼2 million hectares of peatlands. Sarawak on the island of Borneo holds the largest share of Malaysia's peatlands, most of which used to be forested^32^. The two rivers Lupar and Saribas in Sarawak enclose a peninsula with protected peat swamp forest that has a peat thickness of up to 10 m (ref. 33). The estimated peat coverage for the Lupar basin is 30.5% and 35.5% for the Saribas catchment^34^.

SE-Asia is subject to the Malaysian–Australian monsoon as a result of the meridional variation of the intertropical convergence zone. During the wet season from October to April, northern air currents laden with moisture from Asia bring heavy rains to the southeastern parts, whereas southern dry air currents from Australia dominate during the dry season from May to September^35^. Precipitation in Pekanbaru, central Sumatra, ranges from 123 mm in July to 312 mm in November, with an annual sum of 2,696 mm (ref. 36). Rainfall in Kuching, Sarawak, Malaysia, is even higher and ranges from 196 mm in June to 675 mm in January^37^ with an annual sum of 4,616 mm.

### Expeditions

**Figure 1:**
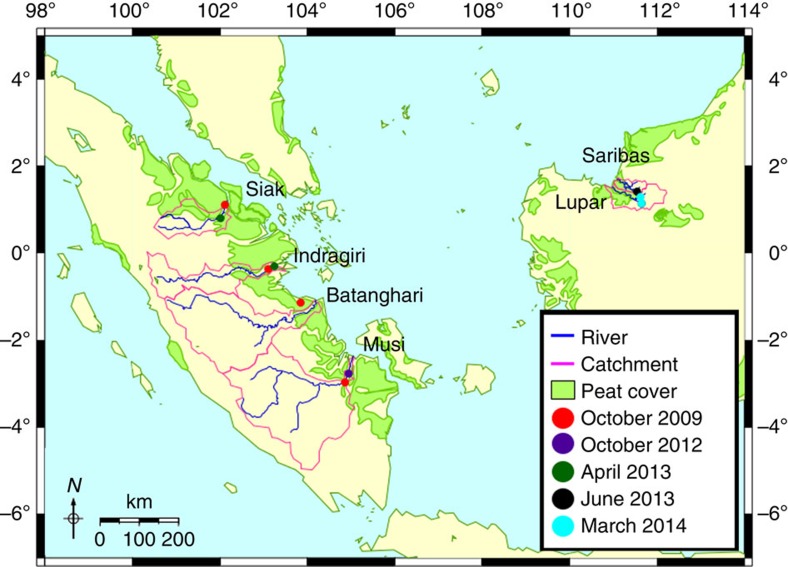
Study area and rivers in Indonesia and Malaysia. The data points indicate the zero-salinity locations in each river, from which the parameter values were averaged.

**Figure 2:**
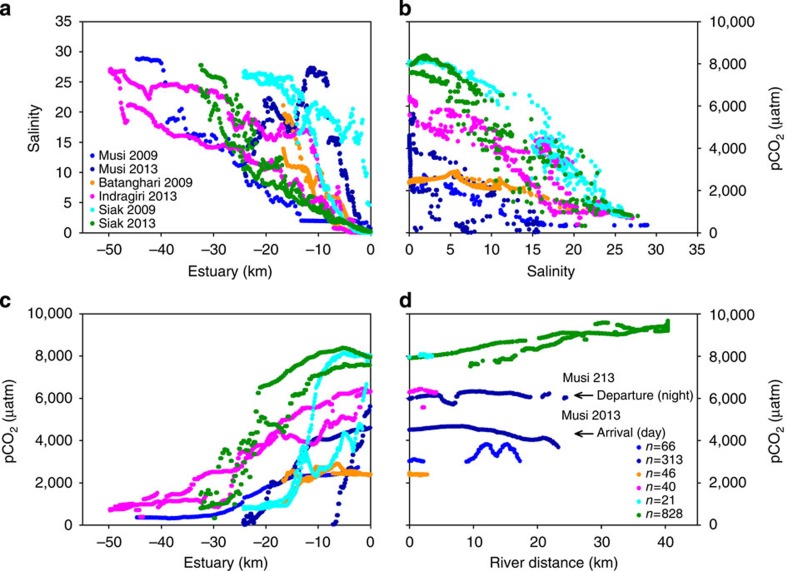
Patterns of salinity and pCO_2_ underway measurements on the way in and out of the estuaries and rivers in 2009 and 2013. Estuaries start at 0 km, which is the border with the river at salinity zero, with increasing (negative) distance towards the coastal ocean. Measurements in the rivers start at 0 km at salinity <0.1 with increasing distance upstream. (**a**) Decrease of salinity across estuary towards river. (**b**) Inverse relationship of pCO_2_ versus salinity (>0.1). (**c**) Increase of pCO_2_ across estuary towards river. (**d**) pCO_2_ data points at zero salinity (0–0.1) against river distance, with number of data points in the lower right corner.

**Figure 3:**
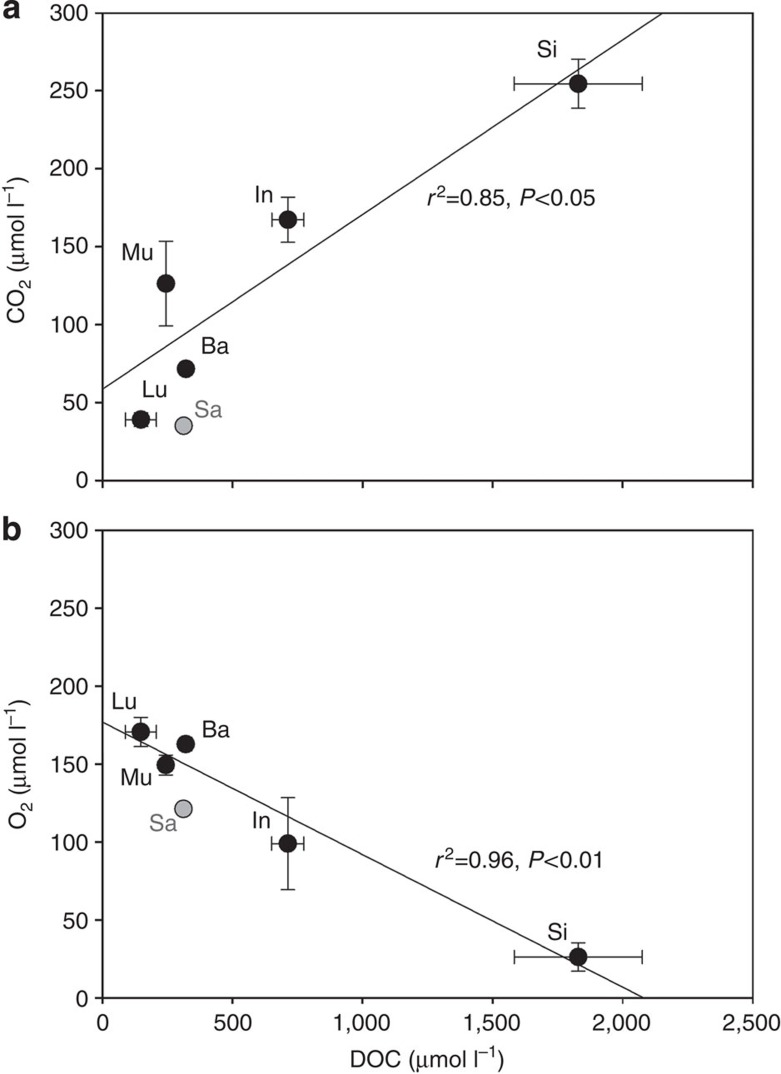
Linear correlations of CO_2_ and O_2_ versus DOC. (**a**) Correlation of CO_2_ versus DOC. (**b**) Correlation of O_2_ versus DOC. All values are in μmol l^−1^. The data points represent annual averages in the Musi, Batanghari, Indragiri and Siak rivers in Sumatra (Indonesia) and the Lupar and Saribas rivers in Sarawak (Malaysia). The Saribas, having only one (seasonal) data point, was excluded from the correlation and is shown in grey. Error bars mean±s.d.

**Figure 4:**
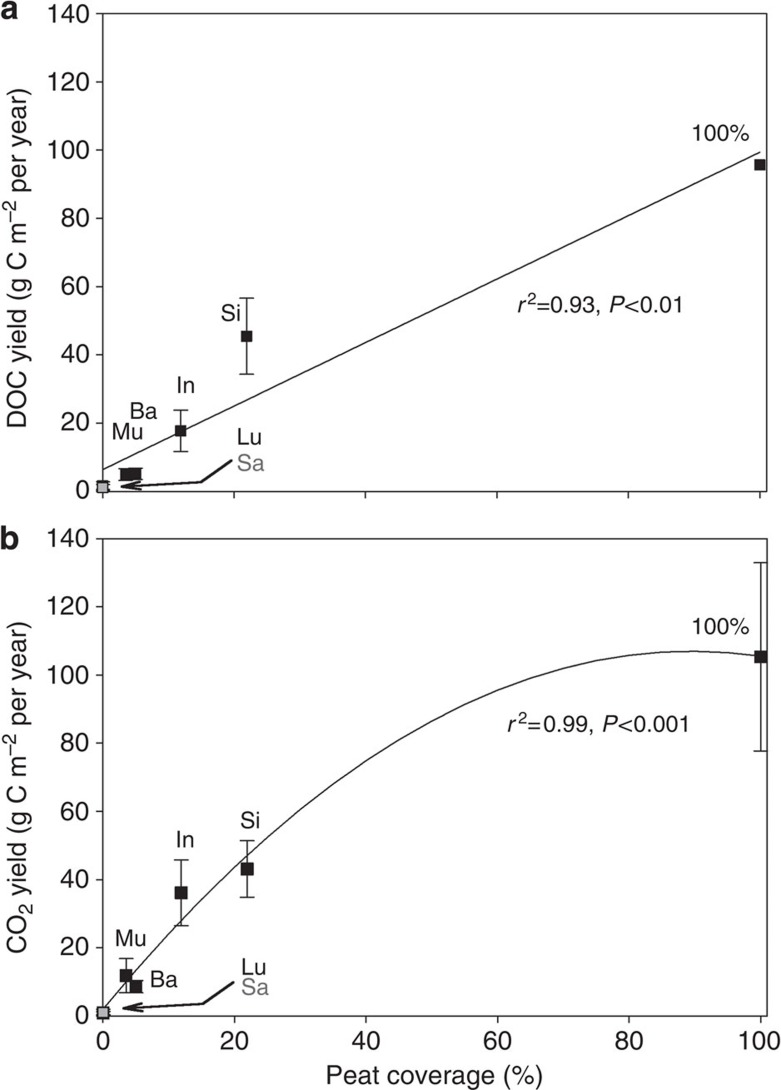
Correlations of DOC yield and CO_2_ yield versus peat coverage. (**a**) Correlation of DOC yield versus peat coverage. (**b**) Correlation of CO_2_ yield versus peat coverage. The yields are shown in g C m^−2^ per year and the peat coverage in percentage. The peat coverage is expressed in percentage for each of the river catchments. The data points represent annual estimates at salinity *S*=0 in the rivers Musi, Batanghari, Indragiri and Siak in Sumatra, Indonesia. The salinity *S*=0 stations in the Lupar and Saribas rivers were located further upstream and outside of the peatland area. Consequently, their DOC concentrations and CO_2_ yields are representative for areas with 0% peat coverage. The Saribas, having only a seasonal data point, was excluded from this correlation, but is shown in grey. Note that the CO_2_ yield is expressed in grams of carbon per m^2^ of catchment area per year, not river area. Error bars represent the best and worst case scenario as a result of incorporating the s.d. of the DOC, pCO_2_, piston velocity and temperature into the DOC and CO_2_ yield calculation.

In this study, data is considered from a total of five expeditions, three of which took place in Sumatra (October 2009, October 2012 and April 2013), and two in Sarawak (June 2013 and March 2014). In October 2009, 72 sampling stations were made and focused mainly on the Siak river and continued along the coast, passing the other rivers to the Musi. In October 2012, the expedition stretched from the Musi river to the Batanghari river with a total of 32 sampling stations. In April 2013 the expedition started in the Musi river going northwest along the other rivers and back via the outer coastal regions, covering a total of 57 sampling stations. In Sarawak, 21 sampling stations were made along the Lupar and Saribas rivers and their estuaries in June 2013 and 26 stations in March 2014. The locations of the river sampling stations at salinity zero are shown on the map in Fig. 1 (ref. 38).

### Sampling methods

In Sumatra, pH, salinity, temperature, dissolved oxygen and pCO_2_ were measured continuously by means of underway instruments. All sensors were arranged in a flow through system and supplied with surface water from an approximate depth of 1 m. Salinity was measured using a Seabird SBE 45 Micro TSG sensor. Temperature and pH were measured with a Meinsberg EGA 140 SMEK with integrated temperature sensor. Oxygen measurements were conducted with an Aanderaa Optode 3835. pCO_2_ was measured with two devices: the Li-Cor 7,000 pCO_2_ analyzer (October 2009 and October 2012) and the Contros HydroC CO_2_ Flow Through sensor (October 2012 and April 2013). Before the expeditions both devices were calibrated, of which the Contros at 100, 448 and 800 p.p.m. The Li-Cor 7,000 analyzer was calibrated with certificated NOAA reference gases (#CB08923 with 359.83 p.p.m., #CA06265 with 1,021.94 p.p.m. and another certified calibration gas with 8,000 p.p.m. CO_2_).

In addition to the continuous measurements, water samples were collected at each station using a Niskin bottle at an approximate depth of 1.5 m. DOC samples were filtered (0.45 μm), stored in 60 ml high-density polyethylene (HDPC) bottles and acidified with phosphoric acid (20%) to a pH-value of 2. After a total storage time during and after the expeditions of maximum 3 weeks, DOC samples were analysed upon arrival in the laboratory in Bremen, Germany, with a Dohrmann DC-190 Total Organic Carbon Analyzer. The samples were combusted at 680 °C within a quartz column, filled with Al_2_O_3_-balls covered with platinum. The released CO_2_ was purified, dried and measured by a non-dispersive infrared detection system. The relative s.d. for the method was ±2%.

In Sarawak, pCO_2_ was measured with an *in situ* FTIR analyzer^39^, using a Weiss equilibrator^40^. In the headwater region, which was not accessible by boat, a Li-820 CO_2_ analyzer was used together with headspace equilibration in a 10-l water bottle (June 2013) and a 600-ml conical flask (March 2014). The FTIR and the Li-820 were calibrated with the same set of secondary standards, ranging from 380 to 10,000 p.p.m. CO_2_. Samples were taken for DOC following the same procedure as described above. Dissolved oxygen, pH and conductivity were measured using a WTW Multi 3420 with an FDO 925 oxygen sensor, a SenTix 940 IDS pH sensor and a TetraCon 925 conductivity sensor. Salinity was calculated from conductivity using the equations by Bennet^41^. Floating chamber measurements were performed to determine the CO_2_ flux. The chamber had a volume of 8.7 l and a surface area of 0.05 m^2^. The CO_2_ flux was determined from the increase of the CO_2_ mixing ratio over time, which was monitored with the Li-820.

### Calibration experiment

To calibrate the Contros river measurements, a CO_2_ calibration experiment was conducted during which different concentrations of CO_2_ gas were delivered using a gas mixing system. Before the calibration with water measurements, the gas concentrations delivered by the gas mixing system were controlled by the mixing system regulator, the Li-Cor 7000, the Li-820 and the cavity ring-down spectrometer (Picarro G2201-i) in a range from circa 500 to 6,000 p.p.m. (Supplementary Fig. 1a). The gas was then used to calibrate freshwater in a range of 1,500–5,500 p.p.m. that was pumped into the Li-Cor 7,000 equilibrator and the Contros sensor. The measured pCO_2_ concentrations were correlated and the regression equation (Supplementary Fig. 1b) was used to calibrate the Contros river data measured during the expedition in 2013.

### CO_2_ flux calculation

CO_2_ fluxes (F) were calculated from pCO_2_ using:


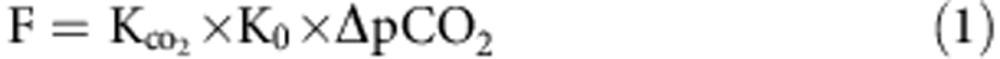


where K_CO2_ is the CO_2_ piston velocity (cm h^−1^), K_0_ the solubility coefficient of CO_2_ in seawater^42^ and ΔpCO_2_ (μatm) is the sea-air pCO_2_ difference.

Usually, the piston velocity is poorly constrained and spatially and temporally highly variable. Raymond and Cole^43^ have pointed out that flux estimates can easily be altered by considerable amounts depending on the choice of the piston velocity. Meanwhile, the ways to determine the piston velocity are multifarious. Relationships with wind speed, hydraulic characteristics^44^, *in situ* measurements using floating chambers^45^,^46^ or dual tracer techniques^47^ are commonly used. Empirical models neglect small-scale fluctuations and allow for estimates on larger scales. These models relate environmental parameters to the piston velocity. In coastal systems and the ocean, this is mostly wind speed^48^. In rivers, stream velocity, stream slope, depth, discharge and bedrock roughness have been identified as the main drivers of in-stream turbulence and consequently the piston velocity^44^. In this study, floating chambers were used to determine piston velocities. The performance of floating chambers is a matter of debate, where both over- and underestimations occur due to artificial turbulence and shielding of wind, respectively, are suggested. On the other hand, sometimes, a relatively good agreement between floating chamber measurements and other techniques is reported^49^. The floating chamber measurements conducted on the Lupar and Saribas river are described in detail in Müller *et al.*^50^. There, we also discuss potential biases. The piston velocities of the Lupar and Saribas rivers were determined with nine floating chamber measurements and are averaged at 26.5±9.3 cm h^−1^ and 17.0±13.6 cm h^−1^, respectively, after normalization to a CO_2_ Schmidt number of 360 (30 °C)^44^. For the Siak river, Rixen *et al.*^10^ derived a similar piston velocity of 22.0 cm h^−1^ using an oxygen balance model. Given that these rivers have piston velocities of a similar range, despite having different catchment sizes^51^ and slopes, we believe that they provide a good representation of the piston velocities in the Indragiri, Batanghari and Musi, Therefore, their average of 21.8±4.7 cm h^−1^ is used to calculate the fluxes in these three rivers, whereas the river-specific piston velocities were applied accordingly in the other rivers.

### Peat coverages

Catchment area was calculated using a relief model of the Earth's surface in ArcGIS 9.3 with the ArcHydro extension. Digital Elevation Data (DEM) such as the SRTM90mDEM can be obtained from the web site of the Consortium for Spatial Information (CGIAR-CSI) of the Consultative Group for International Agricultural Research (CGIAR, http://www.cgiar-csi.org)^52^. The SRTM90mDEM has a spatial resolution of 90 m at the equator and was originally produced in the framework of the NASA Shuttle Radar Topographic Mission (SRTM). Peat coverage (%) for each catchment was estimated using a combination of the FAO Soil Map of the World^31^ and the catchment area derived from the ArcGIS DEM model.

The peat coverage for Malaysia, Indonesia and SE-Asia was derived from Hooijer *et al.*^27^, who based their peat coverage percentages for these areas on field surveys provided by Wetlands International and the FAO Digital Soil Map of the World^31^, as well as Miettinen *et al.*^28^, who used satellite images from as recent as 2010. However, data of Miettinen *et al.*^28^ only covers Malaysia, Sumatra and Kalimantan, whereas that of Hooijer *et al.*^27^ covers entire SE-Asia. Therefore, peat coverage for Malaysia, Indonesia and SE-Asia is based on the combination of Hooijer *et al.*^27^ and Miettinen *et al.*^28^, wherein the most recent data was integrated where possible.

### River surface coverage

For all six rivers, surface area estimation has been conducted based on the length and width of their primary course and main tributaries. Length was estimated using HydroSheds stream lines and Google Earth was used to estimate width along multiple sections in the primary course and main tributaries for each river. River area (%) was calculated based on the share of river surface area with respect to the catchment area. River coverages for Malaysia, Indonesia and SE-Asia are derived from the average pCO_2_, piston velocity and CO_2_ yield found for these locations (Table 6). The results are summarized in Table 2.

### Uncertainty estimates

The errors^53^ associated with the averaged parameter per expedition are presented as the s.d. The errors of the averaged parameter per river are calculated as the s.e. where possible; otherwise the error is presented as the deviation of the two averages from the mean. Throughout the calculations of the CO_2_ yield and fluxes, the s.e. as derived from the averages were integrated. Therefore, the errors of the CO_2_ yields and fluxes are representative for the best and worst case scenarios, named as the best/worst case deviation.

## Additional information

**How to cite this article:** Wit, F. *et al.* The impact of disturbed peatlands on river outgassing in Southeast Asia. *Nat. Commun.* 6:10155 doi: 10.1038/ncomms10155 (2015).

## Supplementary Material

Supplementary Information Supplementary Figure 1

## Figures and Tables

**Table 1:** Averaged parameters per river and year.

**River**	**Time**	**pCO**_**2**_ **(μatm)**	**DOC (μmol l**^**−1**^**)**	**O**_**2**_ **(μmol l**^**−1**^**)**	**Temperature (°C)**	**pH (–)**	Precipitation* **(mm)**
Musi	October 2009	3,388±294	223.3±4.5	158.4±24.9	30.8±0.11	–	197±50
	October 2012	–	264.5±5.3	137.1±48.3	31.4±0.09	6.86±0.25	249±63
	April 2013	5,244±892	–	152.8±55.9	29.7±0.57	–	238±61
	**Average**	**4,316±928**	**243.9±20.6**	**149.4±6.4**	**30.6±0.50**	**6.86±0.25**	**228±34**
							
Batanghari	October 2009	2,400±18	363.0±2.7	162.8±1.3	29.8±0.08	7.07±0.01	180±46
	October 2012	–	286.9±2.8	–	29.5±0.05	–	293±75
	April 2013	–	314.2±5.1	–	30.8±0.05	–	252±64
	**Average**	**2,400±18**	**321.4±22.6**	**162.8±1.3**	**29.8±0.39**	**7.07±0.01**	**242±72**
							
Indragiri	October 2009	5,275±0	774±5.1	128.4±0	29.8±0.00	–	227±58
	April 2013	6,278±206	651.1±4.5	69.4±4.0	32.3±0.13	6.30±0.05	332±85
	**Average**	**5,777**±**527**	**712.6**±**61.5**	**98.9**±**29.5**	**31.1**±**1.25**	**6.30**±**0.05**	**280**±**93**
							
Siak	March 2004†	–	1,866±–	–	–	–	–
	September 2004†	–	2,195±–	–	–	–	–
	August 2005†	–	2,247±–	–	–	–	–
	March 2006†	–	1,613±–	–	–	–	–
	November 2006†	–	1,793±–	–	–	–	–
	October 2009	8,027±40	2,453.0±49.1	17.1±1.2	29.8±0.00	4.78±0.03	318±81
	April 2013	9,083±567	636.1±3.9	35.3±18.3	30.1±0.57	5.48±0.20	206±67
	**Average**	**8,555**±**528**	**1,829.0**±**245.6**	**26.2**±**9.1**	**30.0**±**0.15**	**5.13**±**0.48**	**262**±**79**
							
Lupar	June 2013	1,527±38	88.5±1.8	161.3±0.8	29.0±0.05	6.70±–	88±22
	March 2014	1,021±357	207.9±1.1	180.1±0.9	28.4±0.05	7.10±0.34	167±43
	**Average**	**1,274**±**148**	**148.2**±**59.7**	**170.7**±**9.4**	**28.7**±**0.30**	**6.90**±**0.28**	**128**±**70**
							
Saribas	June 2013	1,159±29	312.2±1.3	121.2±0.6	29.2±0.05	7.30±–	88±22
		**1,159**±**29**	**312.2**±**1.3**	**121.2**±**0.6**	**29.2**±**0.05**	**7.30**±–	**88**±**22**

The spread of the data per individual year is determined by the s.d. The spread of the averages are based on the s.e. if possible, otherwise as the largest deviation from the mean.

^*^Precipitation and s.d.'s derived from Deutsche Wetterdienst (DWD)^54^ and Schneider^55^, respectively.

^†^DOC values derived from Rixen *et al.*^10^. Averaged and used values are in bold.

**Table 2:** River surface area.

**River**	**Length (km)**	**Width (m)**	**Catchment area (km**^2^**)**	**River area (km**^**2**^**)**	**River coverage (%)**
Musi	780	312	56,931	243	0.43
Batanghari	678	374	44,890	269	0.60
Indragiri	366	475	17,968	174	0.97
Siak	447	182	10,423	81	0.78
Lupar*	246	758	6,541	186	0.12
Saribas*	176	444	2,149	78	0.26
					
*Location*†					
Malaysia	–	–	327,291	2,291	0.70
Indonesia	–	–	1,919,317	15,354	0.80
SE-Asia	–	–	2,652,370	20,688	0.78

^*^For the CO_2_ calculations of Lupar and Saribas, only the catchment area upstream from the measurement point was considered, as the measurement data is representative for the upstream river characteristics and not the peatland area further downstream. The river coverage of the entire catchment area of the Lupar is 2.85% and that of the Saribas 3.64%.

^†^River coverages for Malaysia, Indonesia and Southeast Asia are derived from the average pCO_2_, piston velocity and CO_2_ yield found for these locations (Table 6).

**Table 3:** CO_2_ flux versus DOC export – river outgassing in percentage.

**River**	**ΔpCO**_**2**_ **(μatm)**	**K**_**CO2**_ **(cm h**^**−1**^**)**	**River area (km**^**2**^**)**	**Discharge (m**^**3**^** s**^**−1**^**)**	**DOC flux (Gg mth**^**−1**^**)**	**CO**_**2**_ **flux (Gg mth**^**−1**^**)**	**Outgassing (%)**
Musi	3,926±928	21.8±4.7	243	3,054±254	23.6±4.0	55.8±23.3	70.3±6.0
Batanghari	2,010±18	21.8±4.7	269	2,556±422	26.0±6.1	32.5±6.7	55.6±0.7
Indragiri	5,387±527	21.8±4.7	174	1,184±313	26.7±9.4	54.2±14.2	67.0±2.0
Siak	8,165±528	22.0±4.7	87	684±76	39.6±9.7	37.5±7.5	48.6±1.1
Lupar	884±148	26.5±9.3	186	197±86	0.92±0.8	0.83±0.0	47.4±21.0
Saribas	769±29	17.0±13.6	78	44±11	0.44±0.1	0.19±0.0	30.6±5.6
Average							**53.3±6.5**

The error of the ΔpCO_2_ is the s.e., the K_co2_ has the largest error from the mean, the discharge has the s.d. and the DOC flux, CO_2_ flux and outgassing errors are based on the best/worst case deviation, with the average having a s.e. Averaged and used values are in bold.

**Table 4:** CO_2_ yields and fluxes.

**Location**	**Peat coverage (%)**	**Catchment area (km**^**2**^**)**	**CO**_**2**_ **yield (g C m**^**−2**^ **per year)**	**CO**_**2**_ **flux (Tg C per year)**	**Peat impact (%)**
Musi	3.5	56,931	11.8±5.0	0.67±0.28	83.5
Batanghari	5.0	44,890	8.6±1.8	0.39±0.08	77.7
Indragiri	11.9	17,968	36.1±9.7	0.65±0.17	95.1
Siak	21.9	10,423	43.1±8.3	0.45±0.09	96.3
Lupar*	30.5	6,541	61.5±13.0	0.30±0.04	97.7
Saribas*	35.5	2,149	69.0±14.6	0.11±0.01	98.1
Malaysia	7.6	327,291	19.1±4.8	6.2±1.6	90.1
Indonesia	11.9	1,919,317	28.1±6.5	53.9±12.4	93.7
SE-Asia†	10.5	2,652,370	25.2±5.9	66.9±15.7	92.8

The errors of the CO_2_ yield and flux represent the best/worst case deviation.

^*^The Lupar and Saribas stations were located upstream from the peatland area; their data are therefore typical for a peat coverage of 0%. With the Lupar pCO_2_ measurements at 0% peat coverage, the correlation between annual CO_2_ yield and peat coverage was derived (Fig. 4b). The Saribas, having only one (seasonal) data point, was excluded from this correlation, but is shown in grey in Fig. 4b. The correlation was then used to estimate the CO_2_ yields and fluxes of the Lupar and Saribas with respect to their peat coverages of 30.5 and 35.5, respectively, in their catchments.

^†^SE-Asia is defined here as Malaysia, Indonesia, Brunei and Papua New Guinea^27^. Their peat coverages and catchment areas are derived from Hooijer *et al.*^27^ and Miettinen *et al.*^28^ Peat coverage and catchment areas of the rivers were derived using an ArcGIS relief model and FAO Soil map of the world (Methods section).

**Table 5:** pCO_2_ concentrations, piston velocities and CO_2_ yields per COSCAT area.

**Row No.**	**Country covered by COSCAT**	**COSCAT Code**	**pCO**_**2**_ **(μatm)**	**K (cm h**^**−1**^**)**	**CO**_**2**_ **yield (g C m**^**−2**^ **per year)**
1	Malaysia/Brunei	1,328	1,1760	39.58	148.10
2	Malaysia/Indonesia	1,329	11,772	36.67	122.90
3	Malaysia/Indonesia	1,335	7,404	65.83	174.00
4	Indonesia	1,330	11,265	40.83	111.00
5	Indonesia	1,333	458	58.33	0.20
6	Indonesia	1,334	10,497	87.08	124.40
7	Indonesia/Papua New Guinea	1,416	292	66.25	−1.70
8	Indonesia/Papua New Guinea	1,401	372	143.33	−3.00
9	Papua New Guinea	1,402	435	31.67	1.00
10	Papua New Guinea	1,403	1,269	64.17	7.30

**Table 6:** Study comparison of averaged pCO_2_ concentrations, piston velocities, CO_2_ yields and estimated CO_2_ fluxes.

**Location**	**Study**	**pCO**_**2**_**(μatm)**	**K (cm h**^**−1**^**)**	**CO**_**2**_ **yield (g C m**^**−2**^ **per year)**	**CO**_**2**_ **flux (Tg C per year)**
Malaysia	Raymond *et al.*^6^	10,312±1781	47.4±11.4	148.33±18.1	48.8±5.9
	*Lauerwald *et al.*^7^	3,188±575	24.6±2.9	15.1±4.9	4.9±1.6
	†This study	4,369±393	21.8±11.5	19.6±4.0	6.2±1.6
Indonesia	Raymond *et al.*^6^	6,009±4554	59.9±11.3	75.4±30.5	144.7±54.5
	*Lauerwald *et al.*^7^	3,188±575	24.6±2.9	17.3±5.6	33.1±10.7
	†This study	5,535±498	21.9±4.71	26.6±4.6	53.8±12.4
SE-Asia	Raymond *et al.*^6^	5,552±3943	63.4±19.6	68.4±24.4	181.5±64.8
	*Lauerwald *et al.*^7^	3,188±575	24.6±2.9	14.4±5.4	44.6±14.4
	†This study	5,155±464	21.8±7.0	24.5±4.4	66.8±15.7

Studies include that of Raymond *et al.*^6^, Lauerwald *et al.*^7^ and this study. Errors indicate the s.e. (ref. 6), largest deviation from the mean (ref. 7) and best/worst case deviation (this study).

^*^CO_2_ yield and fluxes for Lauerwald *et al.*^7^ are estimated based on the river surface areas defined in our study (Table 2).

^†^Errors for pCO_2_ are based on the average s.e. of 9% as found for the studied rivers.
