# Supplementary material for: The impact of disturbed peatlands on river outgassing in Southeast Asia
Source: Nat Commun. 2015 Dec 16;6:10155. doi: 10.1038/ncomms10155 (PMC4703856; doi:10.1038/ncomms10155)
Supplement: Supplementary Information — Supplementary Figure 1 [file ncomms10155-s1.pdf]

a

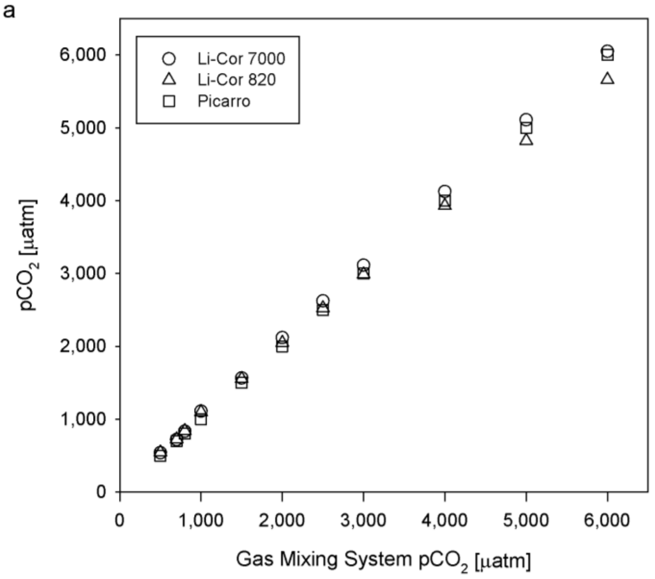

b

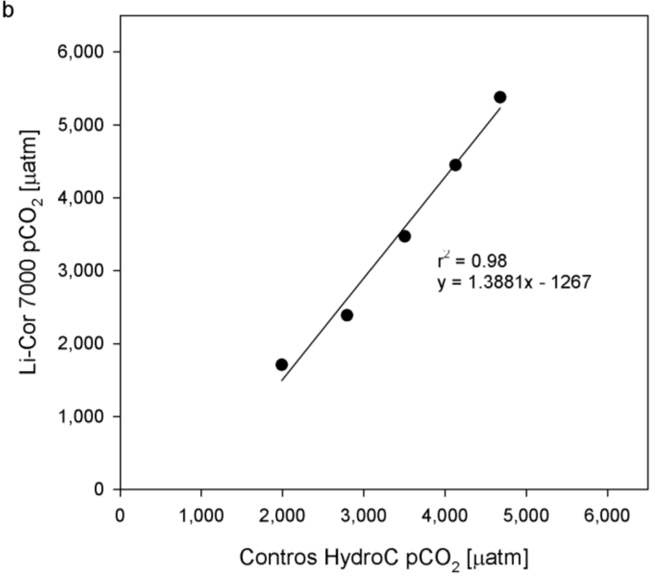

Supplementary Figure 1: Calibration experiment results. a) Gas control with the Li-Cor 7000, Li-820 and Picarro. b) The data points for the Li-Cor 7000 and Contros HydroC can be fitted with a linear regression with $r^2 = 0.98$. The Contros HydroC data was corrected according to this fit.
